# Astrocytic HIV-1 Nef Expression Decreases Glutamate Transporter Expression in the Nucleus Accumbens and Increases Cocaine-Seeking Behavior in Rats

**DOI:** 10.3390/ph18010040

**Published:** 2025-01-01

**Authors:** Jessalyn Pla-Tenorio, Bethzaly Velazquez-Perez, Yainira Mendez-Borrero, Myrella L. Cruz, Marian T. Sepulveda-Orengo, Richard J. Noel

**Affiliations:** 1 Department of Pharmacology and Physiology, Drexel University College of Medicine, Philadelphia, PA 19102, USA; jgp74@drexel.edu; 2 Department of Basic Sciences, Ponce Research Institute, Ponce Health Sciences University, Ponce, PR 00716, USA; bvelazquez@psm.edu (B.V.-P.); ymendez21@stu.psm.edu (Y.M.-B.); mlcruz@psm.edu (M.L.C.); msepulveda@psm.edu (M.T.S.-O.); 3 Department of Biomedical Sciences, Pontifical Catholic University of Puerto Rico, Ponce, PR 00717, USA

**Keywords:** HIV-1 Nef, cocaine, glutamate, nucleus accumbens, GLT-1, xCT, sex-differences

## Abstract

Background/Objectives: Cocaine use disorder is an intersecting issue in populations with HIV-1, further exacerbating the clinical course of the disease and contributing to neurotoxicity and neuroinflammation. Cocaine and HIV neurotoxins play roles in neuronal damage during neuroHIV progression by disrupting glutamate homeostasis in the brain. Even with combined antiretroviral therapy (cART), HIV-1 Nef, an early viral protein expressed in approximately 1% of infected astrocytes, remains a key neurotoxin. This study investigates the relationship among Nef, glutamate homeostasis, and cocaine in the nucleus accumbens (NAc), a critical brain region associated with drug motivation and reward. Methods: Male and female Sprague Dawley rats were used to compare the effects of astrocytic Nef and cocaine by molecular analysis of glutamate transporters, GLT-1 and the cysteine glutamate exchanger (xCT), in the NAc. Behavioral assessments for cocaine self-administration were used to evaluate cocaine-seeking behavior. Results: The findings indicate that both cocaine and Nef independently decrease the expression of the glutamate transporter GLT-1 in the NAc. Additionally, rats with astrocytic Nef expression exhibited increased cocaine-seeking behavior but demonstrated sex-dependent molecular differences after the behavioral paradigm. Conclusions: The results suggest that the expression of Nef intensifies cocaine-induced alterations in glutamate homeostasis in the NAc, potentially underlying increased cocaine-seeking behavior. Understanding these interactions better may inform therapeutic strategies for managing cocaine use disorder in HIV-infected individuals.

## 1. Introduction

HIV-1 remains a global epidemic that affects millions worldwide. In the United States alone, approximately 35,000 contract HIV yearly [1]. Prior to antiretroviral therapy, HIV infection caused early death due to progression to AIDS; current therapeutic advances allow HIV-infected individuals a life expectancy approaching that of the non-infected [2]. Despite this great progress in the clinical management of HIV-1, new complications arise due to the chronicity of the infection [3]. In particular, it has increased the prevalence of HIV-associated neurocognitive disorders (HANDs), a variety of conditions impairing cognitive function that arise due to the effects of the neurotropic virus in the central nervous system [4,5]. To effectively address these challenges, it is important to study their interaction with other common public health issues in this affected community and how they can overlap to further alter neural function.

Substance use disorder is a major public health issue that intersects with HIV-1 infection and clinical management. Drug use has had a significant role in the global spread of HIV [6]. Cocaine use prevalence is higher in individuals who contract the virus, estimated to range from 5% to 15% of HIV individuals, as stated across multiple studies [7,8,9], which could be partially explained by the promotion of high-risk behavior due to drug effects [10,11]. Not only does cocaine use disorder increase the chances of acquiring the virus, but it also hastens the progression of the disease by altering the body’s immune response as well as affecting adherence to medication [12]. Thus, the comorbid use of cocaine in HIV-infected individuals can contribute to the vulnerability to and development of HANDs and can make it difficult to treat and overcome the substance use disorder itself [12,13].

This study explores the complex network of interactions among cocaine use disorder, HIV-1, and glutamate homeostasis in the nucleus accumbens (NAc), a brain structure central to motivation, reward, and addiction. Neuronal homeostasis and function depend on astrocytes, the most prevalent type of glial cell in the CNS [14,15]. Astrocytes are essential for preserving the microenvironment, controlling ion and neurotransmitter levels, and maintaining the blood–brain barrier [15]. Astrocytes play a crucial role in regulating glutamate levels in the brain by up-taking excess glutamate from the synaptic cleft, preventing excitotoxicity, and converting glutamate into non-toxic substances or recycling it to presynaptic terminals [14]. Astrocytes specifically manage glutamate levels via transporters, including EAAT2/GLT-1 and the cysteine–glutamate exchanger (system Xc-), a cysteine glutamate transporter in charge of regulating levels of extracellular glutamate [16]. Glutamate itself plays an important role in synaptic transmission, memory, learning, and neural communication. In the NAc, glutamate plays a key role in mediating reward and pleasure, influencing motivation, and contributing to addictive behaviors [17].

Astrocytes are susceptible to HIV-1 infection and produce early viral proteins, the most abundant of which is Nef [18,19]. Antiretroviral therapy does not target HIV-1 transcription and, thus, does not prevent Nef synthesis by infected astrocytes [20]. The intracellular expression of Nef has been found to interact with signaling pathways, leading to oxidative stress, triggering inflammation, increasing neural degeneration, and dysregulating astrocyte function [18,21,22,23,24]. Moreover, the presence of Nef has been shown to decrease glutamate clearance, leading to excitotoxicity [18,25]. These myriad interactions disrupt homeostasis and contribute to the overall neurotoxicity of the virus [18].

Cocaine increases the expression of GFAP in astrocytes, suggesting that cocaine also plays a role in promoting astrogliosis and disturbing brain homeostasis [26]. In the NAc, cocaine use results in synaptic potentiation and stimulates glutamate release brought on by conditioned signals and drug exposure [26,27]. Disruption of glutamate transporters also increases glutamate access to postsynaptic receptors and potentiates synaptic plasticity that underlies cocaine-seeking behavior [17,26,27].

Glutamate homeostasis is tightly regulated by a family of transporters, and alterations to this equilibrium have been linked to several neurological and psychiatric diseases, including addiction [14]. As described above, HIV-1 Nef and cocaine use are known to independently disrupt glutamate homeostasis, leading to excess extracellular glutamate [17,18,25,26,27,28]. Despite this knowledge, there is a lack of research regarding the possible role of glutamate at the intersection of HIV-1 Nef neurotoxicity and cocaine. Therefore, in this study, the aim is to understand the intricate interplay through which HIV-1 Nef and cocaine can disturb this homeostasis and contribute to the higher prevalence of cocaine addiction in individuals living with HIV. The hypothesis is that HIV-1 Nef expressed in astrocytes of the NAc would cause higher cocaine-seeking behavior in rats through changes in glutamate regulation and stronger synaptic transmission in the reward pathway. A better understanding of the intersection between cocaine and HIV-1 neuropathogenesis can inform therapeutic approaches to HIV-infected cocaine users.

## 2. Results

### 2.1. Astrocytic Nef and Cocaine Had Independent Effects on Glutamate Transporter Expression in the NAc

**Figure 1:**
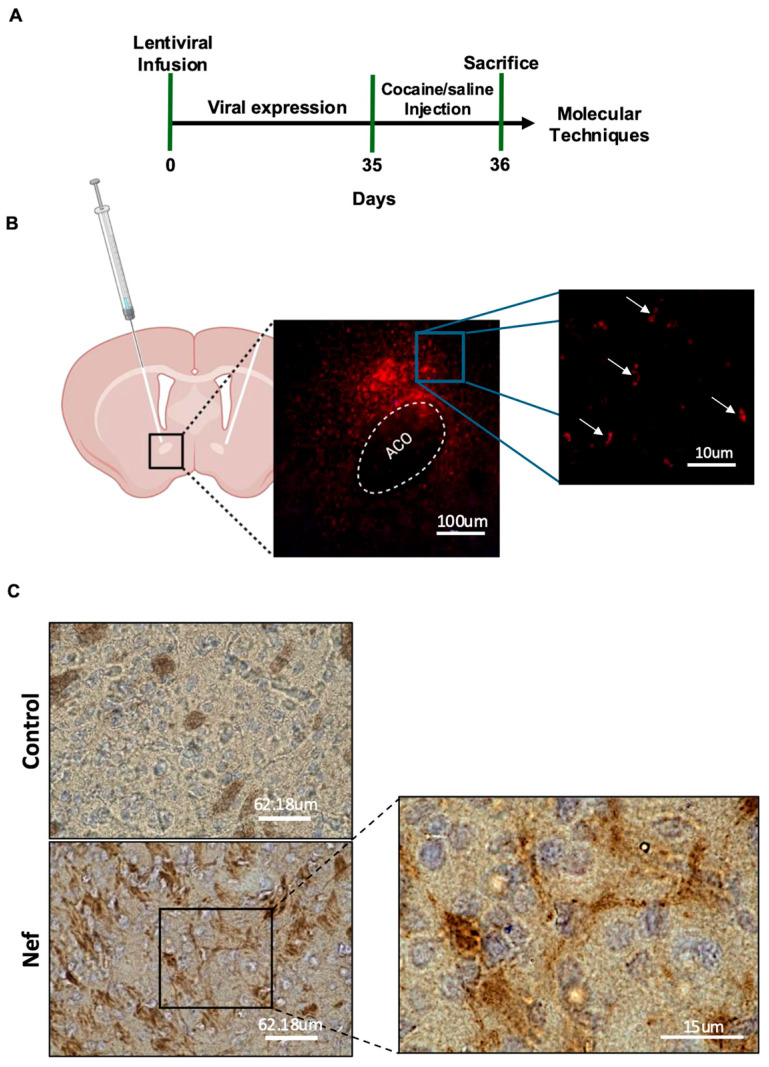
Timeline and schematic representation of bilateral infusions in the NAc at 5 weeks. (**A**) Specific timeline used for molecular experiments with rats. (**B**) Schematic representation showing location of the bilateral infusion site on the corresponding coronal section (red dots). Viral expression spans from approximately bregma 0.70 mm to 1.60 mm. GFAP-specific promoter expression of mCherry in NAc of Sprague Dawley rats 5 weeks after infusion; 10× magnification (100 µm scale). Right image shows white arrows pointing at positive punctate cytoplasmic staining in the NAc (right hemisphere); 100× magnification (10 µm scale). (**C**) Representative images of immunohistochemical staining with Nef antibody (1:50) in NAc slice of Control (top) and Nef-treated (bottom) rat brain tissue. DAB staining in brown with hematoxylin for nuclear counterstain. Pictures of 30 µm thick tissues taken at 60× magnification (62.18 µm scale). Zoomed area at 100× magnification (15 µm scale) of Nef-positive NAc slice demonstrating DAB staining with an astrocyte morphology.

The first part of this study tested the hypothesis that with Nef present, one injection of cocaine is enough to produce strong neurophysiological changes in the NAc and alter the glutamate cycle. To address this question, a lentiviral vector expressing Nef or mCherry was infused into the NAc core of Sprague Dawley rats by bilateral stereotaxic surgery (Figure 1A). After 5 weeks, the infusion site and expression were verified in all rats. The control vector positively expressed mCherry only in astrocytes using the cell-specific GFAP promoter. Figure 1B shows a punctate mCherry expression pattern in astrocytes of the NAc core but not in areas outside of the infusion. Nef expression was confirmed by immunohistochemical staining, which showed positive DAB staining in astrocytes of the NAc core (Figure 1C).

**Figure 2:**
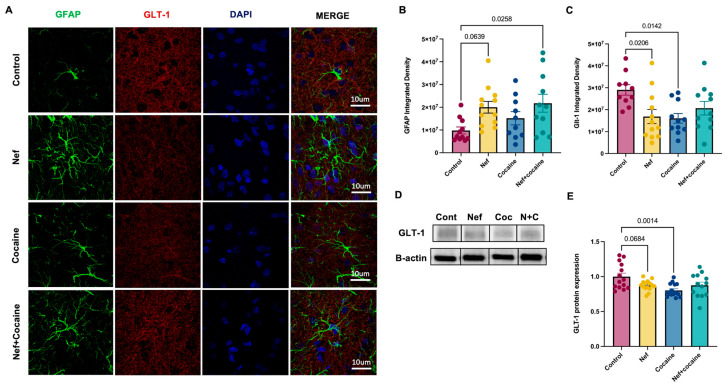
Nef expression in the NAc combined with cocaine causes an increase in GFAP. (**A**) Representative images of free-floating immunofluorescence to identify astrocyte expression using GFAP (green, 1:200) and GLT-1 (red, 1:400). Nuclear counterstaining was used DAPI (blue). Pictures were taken at 100× magnification (10 µm scale). (**B**) Integrated density of GFAP in the infusion site demonstrates increased expression in the Nef+cocaine treatment. (**C**) Integrated density of GLT-1 in the infusion site shows decreased expression after 24 h of cocaine injection (15 mg/kg, i.p.) in Nef and cocaine treatments compared to control. Each sample represents an average of 3–4 60×mag fields per rat in each treatment. (**D**) Representative protein bands of GLT-1 (65 kD) and *β*-actin (42 kD). From left to right: control, Nef, cocaine, Nef+cocaine. (**E**) Protein expression of GLT-1 shows decrease 24 h after cocaine injection (i.p.). Ordinary one-way ANOVA with Tukey post-hoc and multiple comparisons was used to compare data within groups (control: *n* = 10–14; Nef: *n* = 12–15; cocaine: *n* = 10–15; Nef+cocaine: *n* = 11–13). Data represent mean +/− SEM adjusted to *β*-actin and normalized to control. One-way ANOVA main effect between treatments *p* < 0.05.

The next experiment tested whether Nef expression in astrocytes alone had direct effects on glutamate transporters and if a single cocaine exposure potentiated this effect. GLT-1 is the most prevalent transporter to take up extracellular glutamate from the synaptic cleft [29]. Immunofluorescence analysis (Figure 2A) revealed that Nef decreases both the expression and intensity of GLT-1 fluorescence in the NAc core. The expression of astrocytes in the area was confirmed using the astrocyte marker, GFAP (Figure 2B), and demonstrated a clear downregulation of GLT-1 expression around reactive astrocytes (Figure 2C). However, while either cocaine or Nef alone decreased GLT-1 expression, the effect was not additive in combination. These immunofluorescence results were confirmed by Western blot analysis, showing a trending decrease in GLT-1 transporter expression in NAc punches with Nef compared to brains without Nef or a single dose of cocaine (Figure 2D,E). The results also demonstrate a significant increase in astrogliosis with Nef+cocaine compared to control and a trending increase in GFAP in the Nef group (Figure 2B). The data demonstrate that astrocytic Nef expression has direct effects on the reactivation of astrocytes in the brain and also on their function. The effects of Nef expression in astrocytes in the NAc also seem to resemble the physiological effects after a first exposure to cocaine.

**Figure 3:**
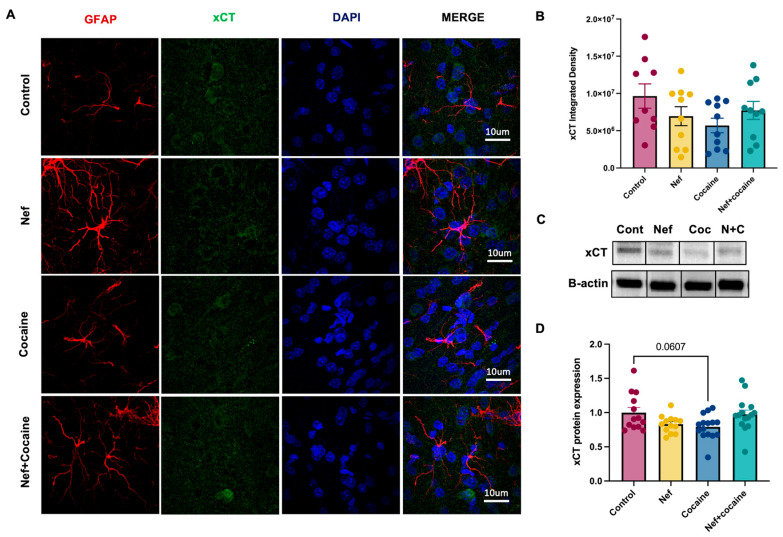
Nef expression in the NAc causes no effect on xCT expression. (**A**) Representative images of free-floating immunofluorescence to identify astrocyte expression using GFAP (red, 1:200) and xCT (green, 1:200). Nuclear counterstaining was used DAPI (blue). Pictures were taken at 100× (10 um scale). Each sample represents an average of 3–4 60× mag fields per rat in each treatment. (**B**) Integrated density of xCT expression in the NAc shows no significant differences between treatments. (**C**) Representative protein bands of xCT (37 kD) and *β*-actin (42 kD) per treatment. From left to right: control, Nef, cocaine, Nef+cocaine. (**D**) Protein expression of xCT in nucleus accumbens shows a decreased trend in cocaine treatment compared to control. Ordinary one-way ANOVA with Tukey post-hoc and multiple comparisons was used to compare data within groups (control: *n* = 9–13; Nef: *n* = 10–13; cocaine: *n* = 10–15; Nef+cocaine: *n* = 10–15). Data represent mean +/− SEM adjusted to *β*-actin and normalized to control.

Expression of the cysteine–glutamate exchanger was also considered due to its role in the astrocyte maintenance of glutamate homeostasis. Past studies have demonstrated that this antiporter, also known as xCT, is also downregulated in astrocytes in response to cocaine consumption [30,31]. Immunofluorescence analysis demonstrated no change in xCT-integrated density after either exposure to cocaine or Nef in the NAc core (Figure 3B). As with GLT-1, no additive effect of cocaine and Nef was observed. Western blot analysis demonstrated only a trend toward a decrease after one dose of cocaine (Figure 3C,D). The data demonstrate that Nef expression in astrocytes in the NAc core does not affect xCT, specifically when measured at 5 weeks post-infusion.

### 2.2. Male and Female Rats with Nef in the NAc Responded Differently to Cocaine Self-Administration

**Figure 4:**
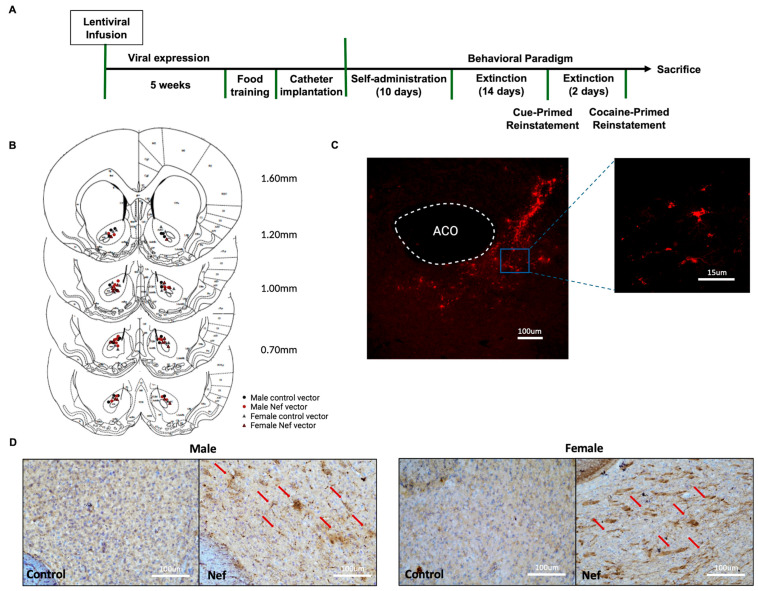
Behavioral timeline and schematic representation of lentiviral infusions in NAc core. (**A**) Behavioral timeline used for cocaine self-administration experiments with rats. (**B**) Schematic representation showing placement of the infusion site of each rat in the corresponding coronal section of the NAc ranging from 0.70 mm to 1.60 mm (male control vectors—black dots; male Nef vectors—red dots; female control vectors—red triangles; female Nef vectors—gray triangles). (**C**) GFAP-specific promoter expression of mCherry in NAc of Sprague Dawley rats 10 weeks after infusion; 10× magnification (100 µm scale) with zoomed-in area at 15 µm scale showing morphology of cells infected. (**D**) Representative images of Nef immunohistochemistry at infusion site after behavioral paradigm. DAB staining in brown with hematoxylin for nuclear counterstain. Red arrows represent astrocytes positive for Nef. Pictures of 30 µm thick tissues taken at 20× magnification (100 µm scale).

Cocaine use prevalence is higher in HIV-infected patients [7,8,9], but few studies focus on the role of early HIV proteins, like Nef, on cocaine-seeking behavior. This study also focused on how HIV-1 Nef can affect drug seeking during cocaine self-administration. Sprague Dawley rats were exposed to a cocaine self-administration paradigm after the expression of Nef by astrocytes in the NAc (timeline shown in Figure 4A). All rats were checked for infusion placement and expression after sacrifice (Figure 4B,C). Nef expression was demonstrated by positive DAB staining in the area of infusion in both male and female Nef+cocaine rats (Figure 4D,E). The Nef-positive cells demonstrated a starlike morphology (shown with red arrows) around the anterior commissure, resembling astrocytes. To reduce the possibility that the sex-based behavioral outcomes could be due to the variability in Nef expression at the infused site, positive DAB staining was analyzed and compared between males and females. No differences in Nef expression were observed between male and female tissues after the behavioral paradigm (Unpaired *t*-test = 0.5454; Supplementary Figure S1).

**Figure 5:**
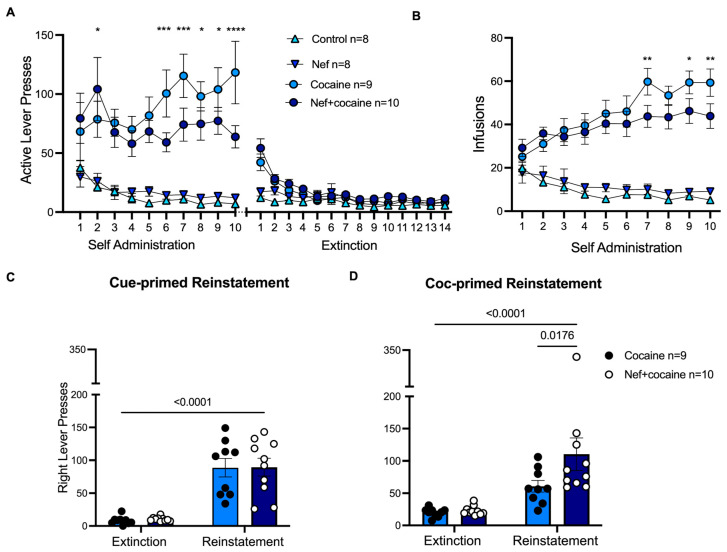
Nef-expressing male rats show more cocaine-seeking behavior than cocaine male rats. (**A**) Active lever presses of control, Nef, cocaine, and Nef+cocaine male rats during self-administration phase and extinction. (**B**) Cocaine infusions of cocaine vs. Nef+cocaine rats and saline infusions for control and Nef rats during self-administration. *, **, ***, **** represent significant differences between cocaine and Nef+cocaine groups during self-administration at *p* < 0.05, 0.01, 0.001, 0.0001, respectively. (**C**) Average right lever presses (correct) during extinction of cocaine vs. Nef+cocaine rats compared to right active lever presses during cue-primed reinstatement. (**D**) Average right lever presses of cocaine vs. Nef+cocaine animals compared to right active lever presses during cocaine-primed reinstatement. Nef+cocaine rats significantly increased active lever presses in cocaine-primed reinstatement compared to cocaine group. Two-way, repeated measures ANOVA with Fisher’s LSD post-hoc analysis indicated all rats reinstated cocaine seeking compared with extinction responding. Data represent mean +/− SEM.

For the behavioral analysis, male and female data were separated because of differences in cocaine consumption. Figure 5 shows male rat behavioral analysis during self-administration, extinction, cue-primed reinstatement, and cocaine-primed reinstatement phases. Male rats expressing Nef had lower active (right) lever presses and, consequently, fewer cocaine infusions during the self-administration sessions than male rats without Nef (Figure 5A,B). During cocaine-primed reinstatement, male rats exposed to Nef significantly increased cocaine seeking compared to the cocaine group (Figure 5D). No differences were observed between cocaine-consuming groups during cue-primed reinstatement (Figure 5C). The data suggest that Nef causes higher cocaine-seeking behavior in male rats, consistent with the reduced cocaine infusions during self-administration; this may be due to higher sensitivity to cocaine when Nef is present in the NAc.

**Figure 6:**
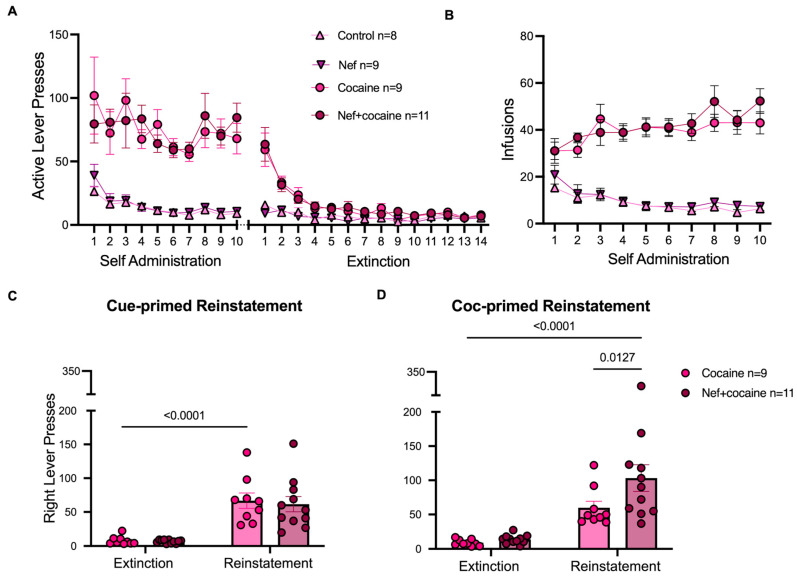
Nef-expressing female rats show more cocaine-seeking behavior than cocaine female rats. (**A**) Active lever presses of control, Nef, cocaine, and Nef+cocaine female rats during self-administration phase and extinction. (**B**) Cocaine infusions of cocaine vs. Nef+cocaine rats and saline infusions for control and Nef rats during self-administration. No differences were observed between the cocaine and Nef+cocaine female groups. (**C**) Average right lever presses (correct) during extinction of cocaine vs. Nef+cocaine rats compared to right active lever presses during cue-primed reinstatement. (**D**) Average right lever presses of cocaine vs. Nef+cocaine animals compared to right active lever presses during cocaine-primed reinstatement. Nef+cocaine rats significantly increased active lever presses in cocaine-primed reinstatement compared to cocaine group. Two-way, repeated measures ANOVA indicated that all rats reinstated cocaine seeking compared with extinction responding. Data represent mean +/− SEM.

The same experimental design was applied to female rats. However, during the self-administration phase, the cocaine and Nef+cocaine female groups had no differences in active lever presses or consumption of cocaine (Figure 6A,B). Like male rats, female rats demonstrated no difference in cocaine seeking during cue-primed reinstatement (Figure 6C) but increased the active lever presses during cocaine-primed reinstatement (Figure 6D). These data, once again, suggest that Nef increases cocaine-seeking behavior when present in NAc astrocytes after short-access cocaine self-administration.

### 2.3. Nef in the NAc Causes Sex-Dependent Differences in GLT-1 and GFAP After Behavioral Paradigm

**Figure 7:**
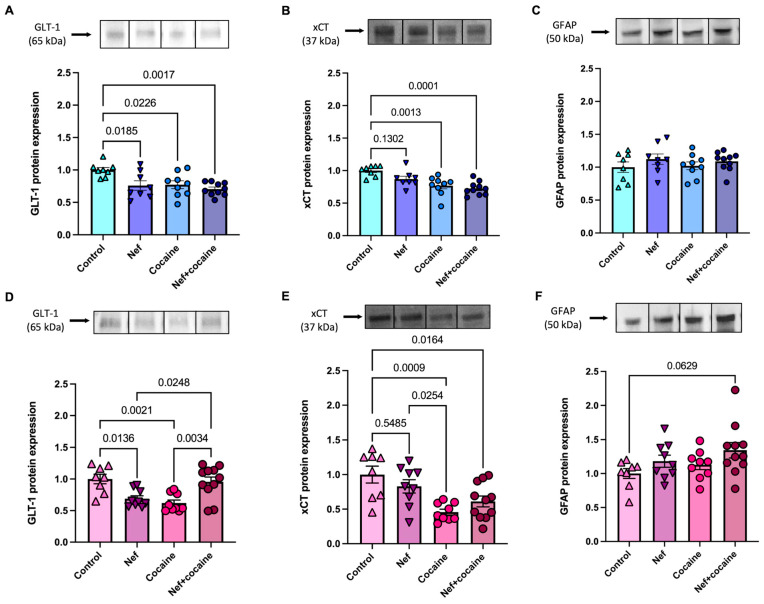
Nef alters glutamate transporter expression after cocaine self-administration paradigm in male and female rats. (**A**) Representative protein bands of GLT-1 (kDa) with analysis of Western blots per group. Protein expression of GLT-1 shows a decrease in all treatments compared to cocaine after cocaine-primed reinstatement of male rats. (**B**) Representative protein bands of xCT (37 kDa) in NAc of male rats with analysis per group. Protein expression of xCT in the NAc shows a decrease in cocaine and Nef+cocaine groups after behavioral paradigm of male rats. (**C**) Representative protein bands of GFAP (50 kDa) in the NAc of male rats with analysis per group. GFAP expression shows no difference between groups of male rats after behavioral paradigm. (male groups, control: *n* = 8; Nef: *n* = 8; cocaine: *n* = 9; Nef+cocaine: *n* = 10). (**D**) Representative protein blots of GLT-1 in the NAc of female rats with analysis per group. Protein expression of GLT-1 shows decrease in Nef and cocaine groups but increase in Nef+cocaine group after cocaine-primed reinstatement in female rats. (**E**) Representative blots of xCT in the NAc of female rats and analysis after behavioral paradigm per group. Protein expression of xCT in the NAc shows decrease in cocaine and Nef+cocaine after behavioral paradigm in female rats. (**F**) Representative images of GFAP protein expression with analysis per group of female rats. GFAP trends toward an increase in the Nef+cocaine female group compared to control 24 h after cocaine-primed reinstatement. One-way ANOVA with Tukey post-hoc and multiple comparisons was used to compare data within groups. (Female groups, control: *n* = 8; Nef: *n* = 9; cocaine: *n* = 9; Nef+cocaine: *n* = 11). Data represent mean +/− SEM adjusted to *β*-actin and normalized to control.

After 24 h of the cocaine-primed reinstatement, rats were sacrificed for the measurement of protein expression of glutamate transporters. NAc punches were collected for protein extraction and Western blotting. Both male and female rats showed decreased GLT-1 expression with Nef or cocaine. However, when cocaine and Nef were combined, males maintained the decrease in GLT-1 expression while females returned to control expression levels. [males: (Figure 7A); females: (Figure 7D)]. In contrast, xCT expression decreased only when cocaine was present, and no differences in males and females were observed [males: (Figure 7B); females: (Figure 7E)]. GFAP expression was also analyzed after the behavioral paradigm to observe if astrogliosis was still present after 10 weeks with the presence of Nef, as observed in previous results. Interestingly, GFAP-only trended towards an increase in the female Nef+cocaine group compared to control, but no differences were found between any of the other groups in male or female rats [males: (Figure 7C); females: (Figure 7F)]. The data suggest an involvement of GFAP expression in the modulation of glutamate transporters, specifically in an apparent neurotoxic environment. Overall, the data suggest that Nef disrupts GLT-1 transporter expression in the NAc and that its regulation is potentially involved in the observed cocaine-seeking behavior during the reinstatement phase.

## 3. Discussion

Despite decades of research, our understanding of the underlying mechanisms driving drug-seeking in SUD remains limited. It is even less clear how the expression of HIV-1 proteins modifies these physiological alterations in the context of SUD in HIV infection. Although antiretroviral therapy (cART) effectively limits HIV replication, astrocytes act as reservoirs in the brain, with latent infection expressing HIV viral proteins, particularly Nef, resulting in inflammation and causing neuronal damage in the CNS [18,32,33]. Cocaine can accelerate HIV infection, increasing viral neurotoxicity by astrocytes and ultimately disrupting neurotransmitter balance in the CNS [13,34,35]. Cocaine directly affects dopaminergic transmission and glutamatergic transmission, both of which are involved in drug-seeking and relapse [36,37,38]. The glutamatergic system plays a significant role in neuroplasticity, drug sensitization, habit formation, and reinforcement learning [37]. Producing HIV-1 neurotoxins in glial cells can alter glutamate homeostasis [35]. Despite this, there is a lack of research regarding the possible role of glutamate at the intersection of HIV-1 Nef and cocaine use disorder. Therefore, this study aimed to address the relationship among Nef, glutamate homeostasis, and cocaine use disorder in the NAc, a critical brain region in motivation, reward, and addiction.

The present study assessed the effects of Nef in the NAc after a single exposure to cocaine or a short-access, long-term exposure to cocaine (self-administration behavioral model). The results demonstrated important molecular and behavioral findings. At a molecular level, the expression of GLT-1 decreased in the NAc after one high-dose exposure to cocaine or astrocytic Nef expression in both male and female rats (Figure 2). The same was found after the self-administration of cocaine repeatedly across 10 days (Figure 7). Interestingly, Nef expression revealed sex-based differences in GLT-1 transporter expression in rats after this self-administration protocol. All rats significantly decreased GLT-1 expression when exposed to Nef or cocaine alone; however, only the male rats maintained this decrease in GLT-1 when Nef and cocaine were combined.

The behavioral experiments showed that Nef expression increased cocaine-primed reinstatement but did not alter cue-primed cocaine seeking in both male and female rats after a short-access self-administration period lasting 10 days (Figure 5 and Figure 6). As with the molecular findings, there were sex-based differences. Only male rats showed reduced cocaine self-administration when exposed to Nef (Figure 5). The data suggest that HIV-1 Nef or cocaine modulates GLT-1 expression in the NAc, while their combination manifests differently in male or female rats (downregulated and unchanged, respectively). These molecular findings do not result in behavioral differences in reinstatement influenced by sex, as results demonstrated that Nef increased cocaine-primed reinstatement in all rats but did not alter cue-primed reinstatement (Figure 5 and Figure 6). Interestingly, Nef expression moderated active lever pressing during acquisition only in male rats, hinting at an increased cocaine sensitivity (Figure 5), and male rats exposed to Nef and cocaine maintained decreased GLT-1 levels, suggesting that reduced glutamate transport may affect sensitivity during self-administration. However, this study was limited in that the design could not assess GLT-1 expression during acquisition, preventing us from determining the timing of the GLT-1 decrease.

### 3.1. Astrocytic Nef and Single Dose of Cocaine Independently Reduce GLT-1, but Not xCT

The glutamate transporter EAAT2, or GLT-1 in rodents, is one of the most abundant proteins in the CNS and is pivotal in maintaining the balance between glutamate release and uptake [34]. It is responsible for 90% of extracellular glutamate reuptake by astrocytes, protecting against excitotoxicity [29,39]. System Xc- is the primary source of extracellular glutamate in a variety of rodent brain areas [40], and it is responsible for up to 60% of extracellular glutamate in the NAc core [31]. The light chain, known as xCT, is primarily responsible for the function of the antiporter [41]. Cocaine use causes changes in extracellular glutamate and glutamate release in different structures associated with the reward system, specifically in the NAc. The NAc is one of the most critical structures to be involved in cocaine seeking. It is composed of glutamate terminals from neurons originating from other reward-related areas, such as the thalamus (Th), amygdala (AMY), hippocampus (HIPP), and prefrontal cortex (PFC) [42]. Therefore, changes in glutamate levels in the NAc have been widely studied during different stages of cocaine administration, including self-administration and single/repeated cocaine doses with/without withdrawal. Reid and colleagues determined that a single high dose of cocaine (15–30 mg/kg i.p) produces an increase in NAc extracellular glutamate of drug-naïve rodents. However, lower doses of cocaine (10 mg/kg i.p. or less) do not acquire the same effect [43,44]. This study successfully demonstrated that one high dose of cocaine (15 mg/kg i.p.) downregulated protein expression of GLT-1 (Figure 2A,C,D), which is consistent with the physiological response of increased extracellular glutamate in the NAc. Nef only showed a significant decrease of GLT-1 by immunofluorescence, meaning a reduced intensity despite no significant changes in total protein by Western blot (Figure 2C). No significant changes were observed in xCT protein expression for any treatment (Figure 3). A possible explanation for the cells to moderate one transporter over the other under these conditions is that more than one cocaine dose is necessary to cause a significant alteration to all transporters and receptors associated with changes in glutamate levels. Also, Nef may not directly affect xCT in astrocytes. More studies may be required to better understand any interaction between xCT and GLT-1 in response to Nef and cocaine.

On the other hand, there are no studies testing glutamate clearance or uptake within an HIV transgenic (Tg) or HIV-1 protein animal model with cocaine. It is known that HIV-1 is associated with neuroinflammation caused by multiple cytokines, such as IL-1β and TNF-α, which can contribute to a reduction in glutamate transporter expression [45,46]. A study in multiple astrocytic cell lines demonstrated that Nef expression led to lower levels of glutamate uptake and release and a higher level of glutamate in the culture media of the infected cells [47]. However, they did not assess glutamate transporter expression. For the first time, this study demonstrated that astrocytic Nef expression in vivo caused a decrease in GLT-1 expression in the NAc and found that one high dose of cocaine (15 mg/kg i.p.) was enough to decrease the expression of this glutamate transporter after 24 h. The downregulation observed could explain the excess extracellular glutamate detected in previous studies. However, the original hypothesis was that astrocytic Nef would exacerbate the rewarding effects of cocaine in the NAc. In this case, differences were observed between Nef and cocaine treatments; when combined (Nef+cocaine), no change was observed when compared to the control group. GLT-1 was only assessed at the time of sacrifice, which is considered a limitation, and the levels could have varied throughout the weeks of Nef expression, cocaine exposure, and behavioral training. That was beyond the scope of this study, so it is challenging to speculate why additive effects were not observed.

### 3.2. GLT-1 Expression Is Reduced by Nef and Cocaine Independently After Cocaine-Primed Reinstatement, While xCT Downregulation Is Cocaine Dependent

The self-administration experiments were focused on discovering what effects astrocytic Nef would cause in the context of cocaine-seeking behavior. Both male and female rats exhibited a higher cocaine-primed reinstatement with Nef in the NAc (Figure 5 and Figure 6). This study extends prior studies with the HIV-1 transgenic rat using a conditioned place preference model that showed potentiated stimulation by cocaine and the HIV transgenes, but only in male rats. A novel aspect of the current study is the limited expression of Nef in the NAc, whereas HIV transgenic expression is not limited to the brain or the reward pathway [48]. Prior work has found increased cocaine sensitivity in male mice expressing HIV-1 Tat protein [49]; however, no data on females was available, as found herein, where Nef shows a sex-dependent effect reducing cocaine self-administration in males only.

Prior studies have predominantly examined dopamine [48], while this study extends to the differences in glutamate transporter expression after re-exposure to cocaine. When astrocytic Nef was present in the NAc of the rats, GLT-1 transporter expression was decreased in intensity (Figure 2C) and expression (Figure 7A). Several preclinical studies have revealed a reduction in GLT-1 expression in the NAc, more precisely in the NAc core, following brief (1 day) and prolonged (40–45 days) periods of cocaine abstinence, along with two to three weeks of extinction, from a typical (2 h per day) cocaine self-administration [50,51,52,53].

Cocaine exposure can reduce levels of xCT, especially in the NAc of rats after self-administration [30,31]. The downregulation of xCT supports drug-seeking behavior for cocaine and other addictive substances by causing glutamate spillover in the NAc core [27]. Additionally, xCT expression is decreased in the NAc core and shell with relapse to cocaine-conditioned place preference (CPP) [54]. To date, no studies have been conducted to assess the effects of Nef and cocaine on glutamate transporter expression. The current study observed differences in xCT expression between cocaine and Nef-treated male and female rats, where the only effect that occurred was cocaine driven after the behavioral paradigm.

### 3.3. Nef and Cocaine in Combination Show Sex-Dependent Effects on GLT-1 Expression After Behavioral Paradigm

Male rats showed a decrease in GLT-1 expression with Nef+cocaine 24 h after cocaine-primed reinstatement, while the female rats exhibited an increase compared to the cocaine group. This difference may be influenced by estrogen receptors expressed on astrocytes. Estrogens have the ability to bind to and activate estrogen receptors (ERs) on astrocytes [55] and can impact astrocyte activity in the brain. Specifically, estradiol, a potent estrogen hormone, has been linked to affecting glutamate metabolism in the brain and plays a protective role against neurodegeneration [56,57,58]. Although studied in cultured astrocytes, researchers have found that estrogen levels positively increase glutamate transporter expression of GLT-1 and GLAST on mRNA and protein levels, decreasing extracellular glutamate levels [56,59]. These results may help explain the increase in GLT-1 expression observed in the NAc of the female rats after the behavioral paradigm as a result of a compensatory mechanism to protect against neurotoxicity. However, the decrease in GLT-1 specifically observed in the Nef+cocaine male rats is thought to be due to sensitivity to the drug. Even if these results were expected, it was not anticipated that there would be a behavioral difference during the self-administration phase. Interestingly, even if Nef+cocaine male rats consumed less than the cocaine group during the acquisition phase, both acquired a similar cue-induced reinstatement after an extinction period. Ample evidence suggests that GLT-1 is downregulated after different withdrawal periods and that a reduction in this transporter, specifically in the NAc, is associated with stronger neuroadaptations linked to cocaine seeking or relapse [50,60,61]. The Nef+cocaine male animals not only consumed less cocaine than the cocaine group, but they later demonstrated a stronger cocaine-induced seeking behavior. The data suggest Nef may induce higher sensitivity to cocaine only in male rats. Additional research should be conducted to clarify the mechanism through which Nef and cocaine are causing sex-dependent behavioral and molecular changes.

### 3.4. Clinical Relevance

Some limitations in the methodology were brought up during the study. First, a low signal for Nef was detected. That may be due to low infection or sensitivity and specificity of the Nef antibody (a known problem in the field). The percentage of Nef expression can be close to 0.1% in astrocytes positive for HIV [62]. A low expression of the protein is expected, which more closely mimics how the actual virus interacts with humans [63]. This limitation did not prevent the detection of differences between treatments and thus was considered minor. However, it is still not known how Nef’s downregulation of glutamate transporter expression in astrocytes involves neuronal changes in the NAc. The focus on one neurotoxin instead of the whole virus is also a limitation but does provide evidence that Nef is sufficient to produce changes in response to cocaine as well as affect proteins responsible for glutamate homeostasis. Clinically, cART effectively lowers HIV viral load to the extent of non-detection, yet no antiretrovirals specifically block HIV transcription. The selection of Nef, an early HIV-1 protein and a potent neurotoxin, builds on prior work, demonstrating that limited HIV-1 expression is sufficient to cause learning impairment [64] and is now shown to alter cocaine-seeking behavior. CNS viral reservoirs persist despite effective cART, raising the prevalence of HANDs in individuals with long-term infection. In patients suffering from severe HANDs, up to 19% of astrocytes test positive for HIV-1. The degree of neuropathological alterations is correlated with the level of infection [19]. Thus, it is essential to determine whether these reservoirs, even when restricted from replicating, may still express viral neurotoxins like Nef that are crucial to the mechanisms behind this comorbidity. While the direct effects of cocaine and Nef protein are not explicitly detailed in the study, the results demonstrate specific interactions through the glutamate system. Understanding the mechanisms by which cocaine and HIV-1 Nef interact could lead to improved and targeted treatments for people living with HIV that use cocaine.

## 4. Materials and Methods

### 4.1. Animals

Adult 60 to 70-day male and female Sprague Dawley rats were obtained from the Ponce Health Sciences University colony. Animals were separated and single housed. Animals were handled for 5 days prior to beginning any procedure and were maintained under standard conditions with a 12 h/light/dark cycle with free access to food and water for Experiment 4.3. Animals for Experiments 4.5 and 4.6 were kept at an inverted 12 h/dark/light cycle with an 18 g food diet. All animal work was approved by the Ponce Health Sciences University Institutional Animal Care and Use Committee (IACUC) and followed the National Institutes of Health’s Guide for the Care and Use of Laboratory Animals.

### 4.2. Bilateral Stereotaxic Surgery

At the time of surgery, adult male rats (~275–310 g) and female (~200–235 g) animals were placed on a stereotaxic apparatus and anesthetized with isofluorane (3–4%). Then, they were stereotaxically microinjected in the targeted area of the nucleus accumbens core, NAc core. Bilateral coordinates (mm) for males: +1.5 anterior/posterior, +2.6 medial/lateral, −7.4 dorsal/ventral and +1.5. anterior/posterior, −2.6 medial/lateral, −7.4 dorsal/ventral. Bilateral coordinates (mm) for females: +1.5 anterior/posterior, +2.6 medial/lateral, −7.2 dorsal/ventral and +1.5. anterior/posterior, −2.6 medial/lateral, −7.2 dorsal/ventral. Infusion was the packaged lentivirus vector of Lenti-GFAP-Nef-IRES-mCherry (Vector Builder cat #LVMP(VB230201-1721wex)-K2) to express HIV-1 Nef and an enhanced mCherry-fluorescent protein driven by the astrocyte-specific GFAP promoter or Lenti-GFAP-IRES-mCherry for controls. Viral vector stocks were independently aliquoted and stored at −80 °C until use and thawed in ice. Virus was microinjected bilaterally to the NAc core using 26-gauge injection cannulas (Plastics One, Roanoke, VA) in a single and dual configuration (1 ul per injection site, 0.05 ul/min injection rate) and left to diffuse for 10 min. After diffusion, the microinjector was slowly removed over 1–2 min, as described previously by Testen et al., 2018 [28]. Then, the incision site was sealed with stitches, and rats were injected with analgesic ketorolac (10 mg/kg i.p.). Animals were provided seven days to recover from surgery and five weeks after infusion to begin experimental procedures. After molecular and behavioral experiments, all infusion sites were histologically confirmed. Animals with improper infusion placements or significant diffusion outside of the target region were rejected.

### 4.3. Cocaine Injection Procedure

Cocaine hydrochloride (provided by the NIDA drug supply program) was dissolved in sterile saline (0.9% solution) to a concentration of 15 mg/mL. For drug administration, animals were injected i.p. at the same time with saline or cocaine. This specific dose is consistent with other literature reviews [65,66,67,68,69]. After 24 h, the animals were anesthetized with an injection of pentobarbital (65 mg/kg i.p.). When the animal was deeply anesthetized (without response to tail pinch), the thoracic cavity was cut with scissors to expose the heart. The right atrium was cut, a dull needle was inserted into the left ventricle, and 20–30 mL of saline solution was perfused to remove the blood from the brain. Immediately following, the animal was decapitated with a guillotine, and the brain was divided in half. The brains were cryopreserved for further molecular analyses of the NAc. Vaginal smears of female rats were taken at sacrifice for corroboration of the estrus cycle phase.

### 4.4. Immunofluorescence

For analysis of GFAP, GLT-1, and xCT expression by immunofluorescence staining, 30 um brain sections were cut in a cryostat (LeicaCM1520) and placed in a 24-well plate, one section per well. Then, sections were washed three times in PBS for 10 min each, followed by two-hour incubation in 10% normal goat serum and 0.3% Triton X-100 in PBS. The tissues were then incubated overnight in primary antibodies GFAP (dil. 1/200, cat#644701, Biolegend), EAAT2/GLT-1 (dil. 1/400, cat#AB1783, Millipore), and SCL711A/xCT (1/200 dil., cat# PA1-16893, Invitrogen), respectively. A tissue section was used as a negative control receiving PBS instead of primary antibody. On the second day, the tissues were washed three times for 10 min in PBS, followed by one-hour incubation in Alexa fluor 488 goat anti-mouse secondary antibody (1/400 dil., cat# A11029, Invitrogen), Alexa flour goat anti-guinea pig secondary antibody (1/400 dil., cat#, supplier) or Alexa fluor 594 goat anti-rabbit secondary antibody (1/200 dil., cat# A11037, Invitrogen). After washing three times with PBS for 10 min each, sections were incubated for 5 min in DAPI (R37606: Invitrogen). Tissues were washed in three intervals of 5 min with PBS buffer and then mounted on slides using ProLong Gold Antifade mountant (P36934, Invitrogen). Three representative areas were photographed for each brain section using a Nikon Confocal microscope at 60× magnification, and the images were further analyzed using ImageJ software version 1.54.

### 4.5. Catheter Implantation Surgery

All rats were given a 5-week recovery period from infusion surgery. After recovery, rats were food deprived for a 24 h period prior to receiving food training sessions in the operant chambers consisting of the acquisition of sucrose pellets through the operant behavior of lever pressing. The operant chamber had two levers: an active lever that provided a sucrose pellet to the rat and an inactive lever that had no effect. Once 100 presses were reached, the rats finished the food training session. After training, animals were exposed to 1.5–2.5% of isoflurane plus Ketorolac (10 mg/kg) as an analgesic for surgical procedures. For implantation of the catheter, a guide cannula (Plastics One, cat# C313G) was attached to a silastic tube (0.025 ID, 0.047 OD Bio-sill), inserted subcutaneously between the shoulder blades, and exited through the skin via a dermal biopsy hole (3 mm). The catheters were then secured by subdermal surgical mesh (Atrium) and the cannula exiting the skin. The other end of the catheter was inserted 3 cm into the right jugular vein and then securely sutured to the underlying muscle tissue. A catheter cap was used when the rats were not connected to infusion pumps. Immediately following catheterization, animals were provided seven days to recover from surgery. After surgery, the IV catheters were flushed daily with 0.1 mL each of 5 mg/mL gentamicin and 70 U/mL heparinized saline for five days following surgery to maintain catheter patency.

### 4.6. Self-Administration Procedure

Patency of the intravenous catheter was maintained by daily infusions of 0.1 mL of heparin saline (70 units/mL) and checked periodically by infusion of 1 mg/0.1 mL i.v. of Propofol (1%; Abbot), a fast-acting anesthetic delivered through the catheter that causes immediate loss of muscle tone and brief sedation (~5 min). Animals were excluded from the experiment if they failed the propofol test. Rats were placed in a self-administration apparatus and allowed to lever press for cocaine on an FR1 schedule of reinforcement (two hours per day for 10 days). Each self-administration cage contained one active and one inactive lever. All sessions would begin and end between the hours of 0700 and 1100 (light cycle). Cocaine (5 mg/mL in 0.9% sterile saline) was housed in a syringe pump external to the training chamber. Each press of the active lever delivered approximately 0.1 mg/kg (in 0.020 mL total volume/infusion for 300 g rat) [64]. A cue light over the active lever was illuminated, and a tone (200 Hz/70 db/5 s) was delivered at the initiation of each infusion. For all experiments, the active lever was deactivated for a 20 s period after each infusion, although all responses on both the active and inactive levers were recorded. The extinction phase consisted of rats being placed inside the apparatus without cocaine infusions, cue light, or tone for 14 consecutive days. A cue-primed reinstatement test was given the day after the last extinction, where rats were re-exposed to the cocaine-associated cues (light and tone) with a right (active) lever press. Two more days of extinction sessions were given between the two tests. For the cocaine-primed reinstatement phase, rats received one dose of 10 mg/kg i.p. cocaine or saline immediately before being placed in respective operant chambers without cues or infusions. Rats were sacrificed 24 h after cocaine-primed reinstatement, as described in a previous section. Vaginal smears of female rats were taken at the beginning of each behavioral phase (SA Day 1 and Ext Day 1), before each test (Cue and Cocaine-Primed Reinstatement), and at the day of sacrifice for corroboration of estrus cycle phase.

### 4.7. Western Blotting

NAc tissue punches were lysed using RIPA buffer of 150 mM sodium chloride, 1.0% NP-40, 0.5% sodium deoxycholate, 0.1% sodium dodecyl sulfate, and 50 mM Tris, pH 8.0. Protein concentration was determined using Micro BCA Protein Assay Kit (Thermo Scientific, Waltham, MA, USA, cat# 23235). An equal amount of protein (25 ug) was assessed by SDS-PAGE in ANY KD gel (Bio-Rad, Hercules, CA, USA, cat# 4569033) and transferred into a polyvinylidene difluoride membrane (PVDF) (Bio-Rad, Hercules, CA, USA, cat# 162-0175). Membranes were blocked with 5% bovine serum albumin (Sigma, St. Louis, MO, USA, cat# B4287) in Tris-Buffered Saline Tween at room temperature for 4 h and incubated overnight at 4 °C with primary antibodies. The following antibodies were used: EAAT2/GLT-1 1:5000 (cat#AB1783, Millipore, Burlington, MA, USA), SCL711A/xCT 1:1000 (Invitrogen, Waltham, MA, USA, cat# PA1-16893), Anti-HIV-Nef monoclonal 1/250 (NIH AIDS Reagent program, Bethesda, MD, USA, cat# 3689), β-actin 1:5000 (Sigma, St. Louis, MO, USA, cat# A5441), GFAP 1:1000 (cat#644701, Biolegend, SanDiego, CA, USA). Then, gels were washed 2 times for 10 min each with TBST and 2 times for 10 min with Blocking solution (non-fat dry milk) and incubated with secondary antibodies, Rabbit 1:5000 (ECL anti-Rabbit IgG, peroxidase-linked species-specific whole antibody, cat# NA934V) or Mouse 1:5000 (ECL anti-Mouse IgG, peroxidase-linked species-specific whole antibody, cat# NA931V), for 1 h at room temperature. Blots were detected using Super Signal Chemiluminescence Substrate (Thermo Scientific, Waltham, MA, USA, cat# 34578) using a ChemiDoc XRS+ Imaging system (Bio-Rad). All Western blots were from separate experiments, each including all four treatment groups to reduce the impact of experimental variability. Quantification of the Western blots was conducted using Image J 1.54d (National Institute of Health, USA). Band densities were normalized to β-actin from the same sample and blot to control for loading. All Western blot data are presented as protein expression compared to control.

### 4.8. Nef Free-Floating Immunohistochemistry

Brain sections were cut at 30 µm thickness with a cryostat (Leica, Deer Park, IL, USA, CM 1520) and placed in a 24-well plate, one section per well. This was followed by a 3% Hydrogen peroxide (Sigma-Aldrich) incubation for 15 min to block endogenous peroxidase and three PBS 1X 3% Triton washes, 15 min per wash. Sections were blocked with normal goat serum (BioGenex, Fremont, CA, USA, cat#HK112-9KE) for 1 h, followed by an overnight incubation with Nef primary antibody (NIH AIDS Reagent Program, Bethesda, MD, USA, cat# 3689; 1/50 dil.). A negative control with PBS instead of primary antibody was run in the procedure. On the second day, the sections were rinsed with three changes of PBS 1X 0.3% Triton for 15 min each, followed by a 4 °C overnight MultiLink secondary antibody incubation (Super Sensitive Link-Label IHC Detection System, cat#LP000-UL, 1/20 dil. in PBS, BioGenex, Fremont, CA, USA). On the third day, the sections were rinsed with three changes of PBS 1X for 15 min each, followed by 2 h Streptavidin Peroxidase (Super Sensitive Link-Label IHC Detection System, cat#LA000-ULE, 1/20 dil. in PBS, BioGenex, Fremont, CA, USA). The sections were rinsed with three washes of PBS 1X for 15 min each, followed by 3,3′-Diaminobencidine (DAB) incubation (BioGenex, cat# HK542-XAKE, Fremont, CA, USA) on each section, and the exposure was monitored for 30 s. Then, the sections were counterstained with hematoxylin and mounted on slides for confocal microscope visualization of Nef expression. Two representative areas were photographed at the high-power field for each tissue for further analysis (Nikon Confocal Microscope, 60× magnification).

### 4.9. Data Analysis

Statistical analysis (summary in Table 1) was performed in GraphPad Prism using a one-way ANOVA with Tukey post-hoc with multiple comparisons analysis to compare groups (Figure 2, Figure 3, and Figure 7). Male and female group molecular analyses of Figure 2 and Figure 3 were combined since no changes were observed between the sexes. For behavioral analysis (Figure 5 and Figure 6), two-way ANOVA with uncorrected Fischer’s LSD post-hoc analysis was conducted to compare groups per day. Values are reported as the mean ± the standard error of the mean (SEM).

## 5. Conclusions

This study provides insights into the complex interactions among HIV-1 Nef, cocaine, and glutamate homeostasis in the NAc of rats. These findings highlight the essential role of HIV-1 Nef and cocaine in glutamate dysregulation, which can relate to higher cocaine use by patients with HIV. Future studies should focus on understanding the mechanism through which HIV-1 Nef disrupts glutamate homeostasis, ultimately altering synaptic plasticity in the context of HIV and cocaine-use disorder.

## Figures and Tables

**Table 1:** Summary of statistical analysis and rationale for use in each figure.

Figures	Statistics	Post-hoc Analysis	Rationale
2, 3, and 7	One-way ANOVA	Tukey’s Post-Hoc with multiple comparisons	One-way ANOVA was used to compare and understand any significant differences between the 4 independent treatments used throughout the study (control, Nef, cocaine, Nef+cocaine). Tukey’s post-hoc analysis also performed pairwise comparisons between the means of each of the groups to identify which of the treatments were different, especially compared to the baseline (control group).
5 and 6	Two-way ANOVA	Uncorrected Fischer’s LSD	Two-way ANOVA was used to analyze the effects of two independent variables (between treatment and phase of behavioral paradigm). Uncorrected Fischer’s LSD was used to focus on a more precise detection of specific contrasts between these variables with a normal distribution within the groups used.

## Data Availability

The raw data supporting the conclusions of this article will be made available by the authors on request.

## Supplementary Materials

The following supporting information can be downloaded at: https://www.mdpi.com/article/10.3390/ph18010040/s1, Figure S1: Quantified Nef expression in NAc between male and female tissues after behavioral paradigm.
