# Supplementary material for: Astrocytic HIV-1 Nef Expression Decreases Glutamate Transporter Expression in the Nucleus Accumbens and Increases Cocaine-Seeking Behavior in Rats
Source: Pharmaceuticals (Basel). 2025 Jan 1;18(1):40. doi: 10.3390/ph18010040 (PMC11769493; doi:10.3390/ph18010040)
Supplement: Supplementary file 1 [file pharmaceuticals-18-00040-s001.zip › pharmaceuticals-3355145-supplementary.pdf]

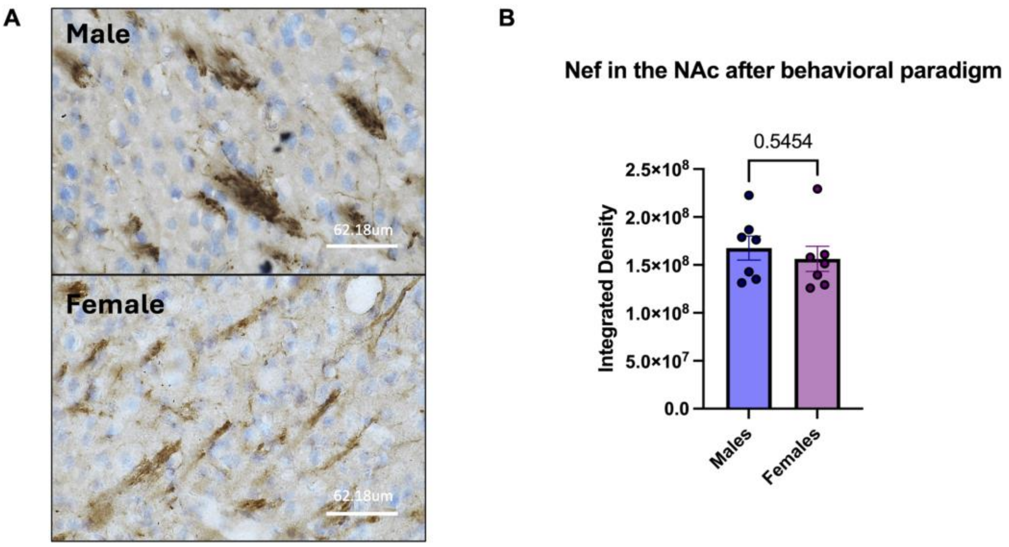

Supplementary Figure 1. Quantified Nef expression in NAc between male and female tissues after behavioral paradigm. (A) Representative images of immunohistochemical staining with Nef antibody (1:50) in NAc slice of male (top) and female (bottom) rat Nef treated brain tissue. DAB staining in brown with hematoxylin for nuclear counterstain. Pictures of 30um thick tissues taken at 60x magnification (62.18um scale). (B) Integrated density of Nef expression in the NAc shows no significant differences between males and females. Each sample represents an average of 4-5 60x mag fields per rat in each treatment. Unpaired T-test was used to compare data within groups (Males: n=7; Females: n=7).
